# News and Notes

**Published:** 1901-10

**Authors:** 


### NEWS AND NOTES.

THE *Atlanta Medical and Surgical Journal* and *Southern Medical Record* having been consolidated into the ATLANTA JOURNAL-RECORD OF MEDICINE, exchanges will please be sent only to the latter. We are getting many duplicates.

DR. W. F. HOLT, a prominent physician and citizen of Macon, is dead.

THE death of Dr. Moncorvo, the well-known pediatrist of Rio de Janeiro, is announced.

AN Egyptian medical congress will be held at Cairo December 10 to 14, 1902, under the patronage of the Khedive.

SAN FRANCISCO is to have a new hospital. It is to be erected by the German General Benevolent Society of that city, at a cost of $250,000.

THE Lonaconing (Md.) Health Board prescribes the killing of flies as a sanitary measure, and an anti-pigpen law will go into effect this fall.

A NEW hospital for tuberculosis is being erected in a suburban section of Hartford, Conn., at a cost of $50,000, half of which has been donated by the State.

THE Health Commissioner of Denver has published a circular requesting victims of tuberculosis to abstain from expectorating on streets and in public conveyances.

THE Board of Health of Manila in a circular letter has called the attention of physicians to the necessity of extreme caution in the differentiation of the ambulatory type of plague.

---

THE term of Surgeon-General W. K. Van Reypen, Chief of the Bureau of Medicine and Surgery in the Navy Department, will expire December 18. Much speculation is being indulged in as to who will be his successor.

---

DR. CALMETTE, the discoverer of antivenine, a snake-bite serum, was recently bitten by a poisonous snake. He gave himself a hypodermic injection of the serum at once, and, although the arm swelled a little and he had a slight fever, he soon recovered.

---

THE *Pester Lloyd* is the authority for the statement that the wife of a Greek priest in Deligrad Servia has just had three girls and three boys born. They are all well-formed and in good condition. Eighteen months ago she had triplets. This makes a total of nine children in a year and a half.

---

MRS. JANE LYNN, of Emlenton, Pa., died recently, says *American Medicine,* after a most remarkable fast. It is reported that in sixty-one days she swallowed only one ounce of food, and that for fifty-five days she took absolutely nothing, with the exception of an occasional glass of water.

---

ENOCH CALLAWAY, A.M., M.D., Jefferson Medical College, Philadelphia, 1876; A.B. and A.M., Mercer University, Macon, Ga., died at his home in LaGrange, Ga., September 21st, aged forty-eight, of Bright's disease. He was a member of the Medical Association of Georgia, mayor of his city for two terms, and was one of the most popular, beloved and successful physicians in Western Georgia.

THE Connecticut Legislature has reported a bill to regulate the practice of osteopathy. It provides for a Board of Examination of three osteopaths, who will examine candidates in anatomy, physiology, pathology, obstetrics, minor surgery, and the principles and practice of osteopathy. No osteopathic physician will be permitted to prescribe drugs or practice surgery.

---

JAPANESE dentists, according to a contemporary, perform all their operations in tooth-drawing with the thumb and forefinger of one hand. The skill necessary to do this is acquired only after long practice, but when once it is obtained the operator is able to extract half a dozen teeth in about thirty seconds without once removing his fingers from the patient's mouth.

---

IN Austria patent medicines and medicinal preparations of which the prescriptions are not open to inspection by physicians, or in which the ingredients cannot be recognized as to quantity and kind, are not allowed to be sold. Every new preparation must be reported to the authorities, and its sale may not be begun until the authorities have found no reason to prohibit the same. Foreign preparations must be accompanied by precise directions for their preparation from the manufacturer. Cosmetics for impure skin, freckles, baldness, etc., are altogether excluded. Prices are regulated according to the value of the ingredients.

---

EXAMINATIONS FOR ARMY MEDICAL DEPARTMENT.—The examination of applicants for appointment as Assistant Surgeon in the Army has been resumed in Washington and San Francisco. The Army Medical Boards convened in those cities will remain in session so long as there are candidates to be examined. Seventy-six vacancies in the Medical Department still remain to be filled, and as it is desired by the military authorities that the department be filled up to its full legal limit as early as practicable, all eligible applicants will be afforded opportunity for examination; those found qualified will be commissioned at an early date. Full information as to eligibility, nature and scope of examination, etc., may be obtained upon application to the Surgeon-General, U. S. Army, Washington, D. C.

